# Increasing Trends of Diabetes Mellitus and Body Weight: A Ten Year Observation at Gondar University Teaching Referral Hospital, Northwest Ethiopia

**DOI:** 10.1371/journal.pone.0060081

**Published:** 2013-03-25

**Authors:** Solomon Mekonnen Abebe, Yemane Berhane, Alemayehu Worku, Shitay Alemu

**Affiliations:** 1 The University of Gondar, College of Medicine and Health Sciences, Gondar, Ethiopia; 2 Addis Continental Institute of Public Health, Addis Ababa, Ethiopia; 3 School of Public Health, Addis Ababa University, Addis Ababa, Ethiopia; 4 Gondar, Ethiopia; Sapienza University, Italy

## Abstract

**Background:**

Diabetes mellitus is becoming one of the major causes of premature adult mortality in developing countries. However, there is a very little documentation of the morbidity trend in such countries.

**Objective:**

To assess the ten-year trend of diabetes mellitus at Gondar University Teaching Referral Hospital, northwest Ethiopia.

**Methods:**

A hospital-based retrospective record review was done at the main referral hospital in northwest Ethiopia. Data were obtained from medical records of all registered diabetic patients in the Diabetic Follow up Clinic between 2000 and 2009. An Extended Mantel-Haenzel chi-square test for the linear trend was used to examine the trend over time.

**Result:**

Out of the total 354,524 patients who visited the Outpatient Department of the hospital during the study period, 1553 (4.4/1000) were diabetes patients, of which 50.1% was type 1 and 49.9% type 2 diabetes mellitus. The average increase in the proportion of both Type 1 and Type 2 diabetes mellitus cases between 2000 and 2009 was 125%. The mean (±SD) age for Type 1 diabetes mellitus was 29.1 (±12), and 53.5 (±12) for Type 2 diabetes. Overall 42.5% of the diabetes mellitus patients were female and 31.7% were rural residents. The mean body mass index for both type of diabetes mellitus increased from 15.9 to 18.3 kg for type 1 and from 23.8 to 24.6 for type 2 between 2000 and 2009, respectively.

**Conclusion:**

The number of diabetes mellitus cases seen at Gondar Referral Hospital is rising steadily. A comprehensive diabetes prevention, treatment, and care program is needed to improve the quality of life of the increasing diabetes mellitus cases in Ethiopia.

## Introduction

Diabetes mellitus (DM) is a chronic condition that stems from the body's inability to produce and/or properly use insulin [1], Over the last 30 years, diabetes has changed from a relatively mild ailment associated with aging to one of the major contemporary causes of premature mortality and morbidity in most countries [2]. Estimates from the 2009 International Diabetes Federation (IDF) suggest that the number of adults living with diabetes in the world will expand by 54%, from 284.6 million (6.4%) in 2010 to 438.4 million in 2030. The projected growth for sub-Saharan Africa (SSA) is 98%, that is from 12.1 million in 2010 to 23.9 million in 2030 [3], [4].

Nearly 80% of these NCD deaths occurred in low and middle-income countries and mortality attributable to diabetes in sub-Saharan Africa in 2010 was estimated at 6% of the total mortality [2], [4]–[6].Currently diabetes affect low-income and developing countries that have the fastest growing numbers of newly diagnosed patients [4].

Ethiopia is one of the top five countries with the highest number of people affected by DM in (SSA)[7]. Previous reports showed that the prevalence of DM in Ethiopia was 2.5% in the year 2000 and was estimated to rise to 3.5% by 2030[8].

Data indicate that proportion of mean body-mass index (BMI), are higher in patients with DM than in those without the disease. Moreover, People who are obese have a risk of type 2 DM of between 5 and 10 times the risk of the non-obese [4]. Thus, DM is a considerable cause of premature mortality, a situation that is likely to worsen and increasingly outstretching the health care resources available in the developing nations [5], [9]–[11].

Data on DM are scarce in Ethiopia. Most clinical data are not timely reviewed and made available for decision makers. This paper tries to show the trend in the burden of DM in the Ethiopian context, which is crucial input for planning health services for the needy and preventing premature adult deaths [10], [11]. Therefore, the objective of this study was to assess the trend of DM over the last ten years at Gondar University Teaching Referral Hospital, which is the only referral treatment center in the northwest part of Ethiopia.

## Methodology

### Study Areas and Population

The study was conducted at Gondar University Referral Hospital in northwest Ethiopia. The actual data sources was the Diabetes Follow up Clinic, which was opened some two decades ago. The hospital is the only referral center in the area and serves in general over 100,000 patients every year in its various general and specialized clinics.

### Study design and data collection

This study was a hospital-based retrospective record review of patients registered at the Diabetic Follow-up Clinic of the hospital from 2000–2009.

A uniform data abstraction sheet was prepared to gather relevant data from the medical records. Data collectors were trained for one day on how to retrieve, abstract relevant data from the medical records, and keep records back in the original location. To ensure the quality of data up to ten percent of the data abstraction sheets were examined by the principal investigator for its competence and consistency.

### Data Analysis

Data were entered on Epinfo version 3.5.3 and analyzed using STATA 11 for Windows 7 software. The key covariate of interest was time (in years) which allowed the estimation of change over time in DM status to estimate the prevalence of DM. Extended Mantel-Haenzel chi-square test for linear trend was used to examine trends in DM proportion for the period 2000 to 2009. The trend was observed by residence, type of DM, and sex.

### Ethical Statement

The protocol was approved by the IRB of the University of Gondar. In addition, a written permit was obtained from the Hospital Director. For the sake of privacy and confidentiality no personal identifiers, such as names, were collected.

## Results

Out of the total 354,524 patients who visited the Outpatient Department of the hospital during the study period, 1553 (4.4/1000) were confirmed diabetic patients. (Table 1) The mean (±SD) age of patients at diagnosis was 41.5 (±17.2), ranging from 4 to 88 years. The mean (±SD) age of patients with Type I DM was 29.7 (±12.6), ranging from 4 to 75 years of age, and of Type II DM was 53.4 (±12); the range was from 14 to 88 years. About 82% of the Type II DM patients were urban residents while about 72% of the rural residents had Type 1 DM, (Table 1).

**Table 1:** Summary results of socio-demographic overall response proportions: Trends of diabetes mellitus in Gondar referral hospital, North West Ethiopia 2000–2009.

Year	Cases	Mean age	Type I DM						Type II DM					
			Residence(#)		Sex(#)		BMI(kg/m^2^)	Total DM(#)	Residence(#)		Sex(#)		BMI(kg/m^2^)	Total DM(#)
			Urban	Rural	M	F			Urban	Rural	M	F		
2000	112	41.3	12	42	36	18	15.9	54	49	9	28	30	23.8	58
2001	75	40.3	9	28	24	13	16.7	37	33	5	23	15	24.4	38
2002	108	38.3	19	40	42	17	16.5	59	43	6	26	23	23.3	49
2003	156	39.9	29	60	65	24	16.4	89	52	15	35	32	23.2	67
2004	203	41.7	24	82	70	36	16.9	106	74	22	50	46	24.9	97
2005	102	36.5	16	48	42	21	16.6	64	30	8	22	16	23.9	38
2006	180	45.1	32	42	44	30	17.9	74	90	16	47	58	24.3	106
2007	196	44.9	23	61	44	40	18.1	84	91	21	61	51	24.3	112
2008	176	42.4	26	55	50	31	18	81	87	8	47	48	24.5	95
2009	245	40.2	27	103	93	37	18.3	130	88	27	55	60	24.6	115
Total	1553	41.5	217	561	510	267	17.3	778	637	137	394	379	24.2	775

BMI- Body mass index, M- Male, F- Female

There were more Type 1 DM patients in rural areas compared to urban residents, and much more Type 2 DM patients were from urban areas compared to rural areas (Table 1).

About 65.6% of the Type 1 DM patients were men while about 51% of the women had Type 2 DM.

The overall increase in the proportion of DM out of total patients seen in the hospital from 2000 to 2009 was 125%. Both Type 1 and Type 2 DM proportions showed increasing trends during the study period Mantel-Haenzel chi-squre test was statistically significant for both Type I and Type II DM (, p<0.0001 (Table 2).

**Table 2:** Trends of DM at Base-line (Extended Mantel-Haenzel chi-square and proportion) North West Ethiopia (2000–2009).

Year	DM patients (#)	OPD Patients (#)	Proportion of DM (%)	95% CI	Mantel-Haenszel OR
2000/1	187	86014	0.22	[0.18–0.25]	1
2002/3	264	65375	0.4	[0.35–0.45]	1.861
2004/5	305	88212	0.35	[0.30–0.38]	1.592
2006/7	376	62643	0.6	[0.54–0.66]	2.771
2008/9	421	52280	0.81	[0.73–0.88]	3.726
Total	1553	354524	0.44		Chi^2^ = 270.35 (P<0.0001)

The lowest mean Body Mass Index (BMI) of 17.3 kg/m^2^ was noted in Type 1 DM, while it was 24.2 kg/m^2^ for Type 2 DM.

**Figure 1:**
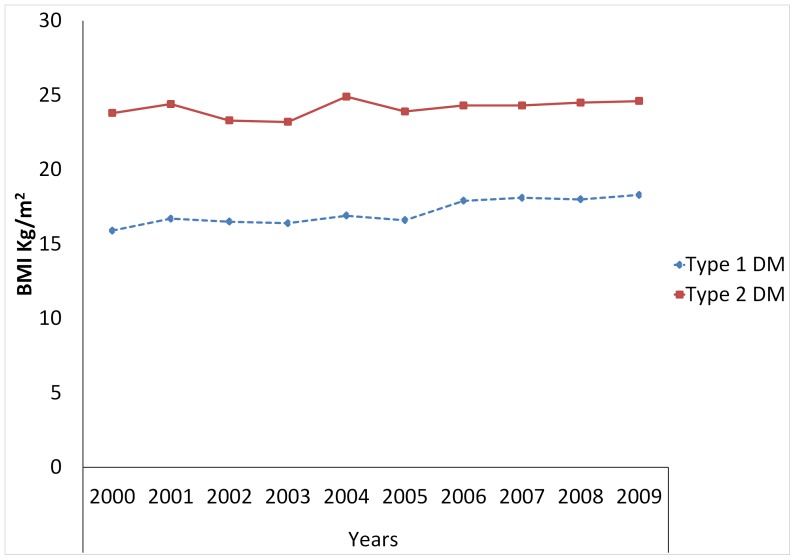
Trends of BMI among diabetic patient in Gondar Hospital, North West Ethiopia: A ten year observation (2000 to 2009).

The mean BMI increased from 15.9 to 18.3 kg for type 1 DM and from 23.8 to 24.6 for type 2 DM between 2000 and 2009, respectively. The mean BMI at presentation showed an increasing trend over time; the linear regression association was statistically significant among type 1 DM (*P*<0.001) (Table 1, figure 1).

## Discussion

Our findings show that the proportion of DM patients has significantly increased over the 10 year observation period (2000–2009) in the study hospital for both Type 1 and Type 2 DM. Similar observations were reported from teaching hospitals in the capital Addis ababa [12], [13]..

These data suggest that the burden of DM is increasing perhaps as a result of demographic changes and also due to better access to the referral hospital. Ethiopia has a policy to treat DM patients free of charge in public health facilities. Thus, unlike other diseases, DM cases are less likely to go to private clinics in the study areas, and self-selection bias is the least concern in this study.

In line with this finding, a study conducted in SSA showed that the mean age for the onset of Type 1 DM was late by more than a decade, compared to that of the European population [14], [15]. This could be due to early exposure to environmental risk factors predominantly seen in urban communities of developed countries.

In our study the mean BMI score at first recognition of type 1 DM was under-weight. A study done in USA shows that patients diagnosed with type 1 diabetes had a lower BMI [16]. In line with this, the WHO disease classification report shows that the disease phenotype encountered in Africa is very different from that of the West, and most closely resembles a malnutrition-related diabetes. The low BMI could partly be due to the loss of weight as a result of consuming their body fat at the time of diagnosis due to delayed detection of the case.

There was also a significant association between the BMI score and the age at diagnosis of type 1 (P<001). Similarly, a study done in USA showed a significant positive correlation between patient BMI score and the age at diagnosis of type 1. The observed increased BMI could be one of the risk factors for the early manifestation of the disease, or other risk factors may play their role for the observed rise [16]–[19].

Moreover, the mean value of BMI at first recognition of type 2 DM was normal weight as per the WHO classification, and it also showed slight increases during the study period. Furthermore reports from Mali, Nigeria, and Tanzania have shown that the prevalence of DM increases with increasing BMI. However, the mean BMI of the study patients was below 25 kg/m^2^: this indicates that the known parameter of overweight/obesity as a risk factor of DM was not significantly associated for this group. More importantly, other risk factors favoring diabetogenesis (more of genetic/family history) might have played a significant role [20].

Maintaining good quality information at diabetic follow up care clinics is essential for a better monitoring of disease trends. One of the limitations of our study was the in-completeness of the data/information; the review charts had some missing data for some variables (<10%), since, the study was based on a retrospective record review. This could have led to differences in unmeasured independent variable for which no correction could be made. We used a list-wise deletion for missing variables such as medication use and diabetic related complication to have a complete case analysis across time. Therefore, there is need for the improvement of documentation and information network.

### Conclusion

The proportion of diabetes mellitus at Gondar Referral Hospital is increasing steadily. This finding calls attention to the urgent need for monitoring non-communicable diseases such as diabetes mellitus in order to facilitate timely preventive and curative interventions.
